# AAV Vectored Immunoprophylaxis for Filovirus Infections

**DOI:** 10.3390/tropicalmed5040169

**Published:** 2020-11-09

**Authors:** Amira D. Rghei, Laura P. van Lieshout, Lisa A. Santry, Matthew M. Guilleman, Sylvia P. Thomas, Leonardo Susta, Khalil Karimi, Byram W. Bridle, Sarah K. Wootton

**Affiliations:** Department of Pathobiology, Ontario Veterinary College, University of Guelph, Guelph, ON N1G 2W1, Canada; arghei@uoguelph.ca (A.D.R.); lvanlies@uoguelph.ca (L.P.v.L.); lsantry@uoguelph.ca (L.A.S.); guillemm@uoguelph.ca (M.M.G.); sthoma13@uoguelph.ca (S.P.T.); lsusta@uoguelph.ca (L.S.); kkarimi@uoguelph.ca (K.K.); bbridle@uoguelph.ca (B.W.B.)

**Keywords:** monoclonal antibodies, adeno-associated virus vector, vectored-immunoprophylaxis (VIP), vector biology, filoviruses, viral hemorrhagic fever, zoonotic diseases

## Abstract

Filoviruses are among the deadliest infectious agents known to man, causing severe hemorrhagic fever, with up to 90% fatality rates. The 2014 Ebola outbreak in West Africa resulted in over 28,000 infections, demonstrating the large-scale human health and economic impact generated by filoviruses. *Zaire ebolavirus* is responsible for the greatest number of deaths to date and consequently there is now an approved vaccine, Ervebo, while other filovirus species have similar epidemic potential and remain without effective vaccines. Recent clinical success of REGN-EB3 and mAb-114 monoclonal antibody (mAb)-based therapies supports further investigation of this treatment approach for other filoviruses. While efficacious, protection from passive mAb therapies is short-lived, requiring repeat dosing to maintain therapeutic concentrations. An alternative strategy is vectored immunoprophylaxis (VIP), which utilizes an adeno-associated virus (AAV) vector to generate sustained expression of selected mAbs directly in vivo. This approach takes advantage of validated mAb development and enables vectorization of the top candidates to provide long-term immunity. In this review, we summarize the history of filovirus outbreaks, mAb-based therapeutics, and highlight promising AAV vectorized approaches to providing immunity against filoviruses where vaccines are not yet available.

## 1. Introduction

Infectious diseases have had profound and long-lasting impacts on the human race throughout history. Epidemic threats are deepened by the emergence of new and uncharacterized infectious diseases, coupled with the ability to impact human health and the economy at a global scale. Although our understanding and surveillance of infectious disease has advanced, the pursuit of effective methods for preventing the spread of these infections at times remains elusive. Filovirus disease outbreaks showcase these concerns, due to their high pathogenicity, zoonotic transmission efficiency, and spontaneity of spillover. Although Ebola hemorrhagic fever (EHF) and Marburg hemorrhagic fever (MHF) are highly pathogenic viral diseases, the global burden of EHF and MHF is minor in comparison to other infectious diseases [1]; however, as we observed with the 2014 West Africa outbreak, EHF has the potential to cause large, multi-nation outbreaks resulting in significant mortality and economic devastation. In this review, we will discuss the history and pathogenesis of filoviruses, highlight the role of antibodies in protection against filovirus infections, and examine the potential of viral vector-mediated expression of monoclonal antibodies (mAbs) as an alternative prophylactic strategy to enable long term passive immunity against filovirus infections.

## 2. Filoviruses

### 2.1. A Brief History of Filovirus Outbreaks

Filovirus outbreaks have been reported since 1967, with the first outbreak of Marburg virus (MARV) occurring simultaneously in Germany and Yugoslavia, when laboratory workers imported African green monkeys (*Chlorocebus aethiops*) from Uganda and were exposed while working with the tissues of infected animals [2]. The second outbreak of Marburg virus disease (MVD) occurred in 1975 in South Africa, where it was determined that the index patient had visited caves in Rhodesia (now Zimbabwe) and had come in contact with bats [3]. A subsequent Marburg outbreak occurred in Kenya in 1987, where the index case had also visited a cave and contracted the virus; however, in this case the disease was caused by a new strain of *Marburgvirus*; Ravn virus (RAVV). In total, there have been 13 recorded MARV outbreaks (MARV and RAVV) with over 460 confirmed cases and 370 reported deaths [4].

Shortly after the discovery of MARV, there were three outbreaks of *Sudan ebolavirus* (SUDV) and *Zaire Ebolavirus* (EBOV) in 1976, across Sudan, the Democratic Republic of Congo (DRC) (formerly Zaire), and England [5]. Since the identification of Ebola virus in 1976, there have been a total of 38 Ebola virus disease (EVD) outbreaks, including the recent EBOV outbreak in the DRC, which was announced 1 June 2020 [6]. The largest filovirus outbreak occurred from December 2013 to March 2016, shedding light on the true epidemic potential of EBOV. Epidemiological and genomic analyses suggest that the index case was a 2-year old boy in Meliandou, Guinea, who had been infected through exposure to bats [7]. By the time multiple cases of fatal diarrhea were reported and the Pasteur Institute had confirmed EBOV was the cause, the disease had already spread to the capital of Guinea, Conakry [8], as well as to neighboring countries, Sierra Leone and Liberia. On 23 March 2014, the WHO officially declared an outbreak of EVD. Inadequate disease surveillance, poor public health infrastructure, the ravages of civil war, extreme poverty, and local customs, such as washing a dead body prior to burial, aided in the spread of EBOV [9,10]. After more than two years, the outbreak was declared over in June of 2016, claiming the lives of more than 11,320 people and infecting a staggering 28,600 individuals [11]. The unprecedented scale of this outbreak left many survivors suffering from post-Ebola syndrome [12], orphaned more than 17,000 children [13], and devastated economies. Moreover, the EVD outbreak reduced the availability of treatments and monitoring for other serious infectious diseases, including HIV, tuberculosis, and malaria leading to increased mortality [14]. Despite the fact that previous filovirus outbreaks had highlighted the potential for efficient transmission and high case fatality rates, there were no U.S Food and Drug Administration (FDA)-approved vaccines or therapeutics for EBOV prior to the 2014 West Africa Ebola outbreak.

### 2.2. Filovirus Taxonomy

Filoviruses are a family of non-segmented, negative-sense RNA viruses belonging to the order *Mononegavirales*. Filovirus taxonomy has been frequently updated during the past decade, where the most recent update by the International Committee on Taxonomy of Viruses in July of 2019 listed six genera and eleven species in the family [15]. The six genera within the Filoviridae family include: *Ebolavirus*, *Marburgvirus*, *Cuevavirus*, *Thamnovirus*, and *Striavirus*, and the most recently classified, *Dianlovirus* [16,17]. The *Ebolavirus* genus contains six species: the highly pathogenic *Zaire ebolavirus* (EBOV, Ebola virus) and *Sudan ebolavirus* (SUDV, Sudan virus), the less prevalent *Taï forest ebolavirus* (TAFV, Taï Forest virus), *Bundibugyio virus* (BDBV, Bundibugyio virus), *Reston ebolavirus* (RESTV, Reston virus), and the recently discovered *Bombali ebolavirus* (BOMV, Bombali virus) [18]. The genus *Marburgvirus* contains a single species, *Marburg marburgvirus* (MARV, Marburg virus); however, two distinct strains with less than 30% genetic divergence, *Marburg virus* (MARV) and *Ravn virus* (RAVV), make up this species [19]. *Lloviu cuevavirus* (LLOV, Lloviu virus), isolated from insectivorous bats located in Northern Spain, is the only species confirmed in the *Cuevavirus* genus [20]. Of the members in the Filoviridae family, seven species have been confirmed to infect humans, including EBOV, SUDV, TAFV, BDBV, MARV, RESTV, and RAVN, albeit with different severity [21]. The most virulent is EBOV, followed closely by MARV with fatality rates ranging from 25–90% and 24–88%, respectively, whereas, BDBV and SUDV are less severe with fatality rates of ~30% and 50%, respectively [21,22,23]. Though RESTV does cause infections in humans, these have only ever been reported as asymptomatic; however, it can be fatal in non-human primates (NHP) [24]. Limited information is known about TAFV infections in humans as there has only been one documented case, with that person recovering after a severe illness [25].

### 2.3. Filovirus Molecular Biology and Pathogenesis

Filoviruses are filamentous enveloped particles 80 nm in diameter and up to 14,000 nm in length [26]. The negative-sense, single stranded RNA genome is approximately 19 kb in length and encodes seven open reading frames (ORF) orientated in a 3′-5′ direction: the nucleoprotein (NP), viral protein (VP) 35, VP40, glycoprotein (GP), VP30, VP24, and the RNA-dependent RNA polymerase (L) [27]. Each ORF is flanked by non-translated regions including conserved transcriptional start and stop signals crucial for protein expression [28,29]. The GP for filoviruses is the only protein “studded” on the surface of the virion and is the sole determinant of viral entry into host cells [30]. In addition to the fundamental role of the GP in viral entry, ebolavirus GPs appear to have multiple auxiliary functions, likely contributing to the complex pathogenesis of the virus [31].

As humans are not the natural reservoir hosts for filoviruses, spillover occurs through contact with the virus’s natural reservoir hosts, which in the case of EBOV is likely to be a species of bat [32]. Alternatively, transmission can occur through contact with intermediate hosts, for instance when hunting bushmeat, or through secondary transmission by infected humans. In patients with disease, acute EBOV and MARV virus shedding occurs and can be found in blood or other bodily fluids including: urine, saliva, sweat, feces, vomit, breast milk, and semen [33]. Once an individual becomes infected there is a 2–21 day incubation period, with calculated mean incubation periods of 5.3–12.7 days for EBOV, 3.35–12 days for SUDV, and 6.3–7 days for BDBV, characterized by onset of non-specific flu-like symptoms [34,35,36,37,38,39,40]. Following this incubation period, disease occurs rapidly in lethal cases, with high fever, severe hemorrhage, shock, followed by death, due to systemic viral replication, immunosuppression, and abnormal inflammatory responses with extensive organ distribution [41,42,43]. Upon entering the host, filoviruses preferentially infect antigen presenting cells (APCs) including dendritic cells (DCs), monocytes, and macrophages [44,45,46,47]. Infected APCs fail to activate and mature and are therefore unable to present antigens to T cells in the lymph nodes. Upregulation of co-stimulatory molecules (i.e., CD40, CD80, CD86, and MHC class II) is inhibited in infected APCs, which subsequently interferes with their ability to initiate adaptive immune responses [48,49]. Additionally, studies have shown that infection of EBOV and MARV results in lymphopenia, affecting CD4+ and CD8+ T cells, as well as B cells and natural killer cells [50,51,52]. Loss of B cells, as well as helper T cells, leads to impairment in humoral responses, as there is an absence of specific IgG and barely detectable IgM in fatal infections [53]. Conversely, Ebola survivors have revealed significant activation of both B and T cells, proliferating plasmablasts, as well as circulating Ebola virus-specific IgG [54,55].

## 3. Filovirus Vaccine Development

After the first Ebola virus outbreak in 1976, research began on an inactivated EBOV vaccine, which was shown to be efficacious in guinea pigs in 1980 [56]. Due to the sporadic nature of the outbreaks and the low number of confirmed cases, development of a vaccine against EBOV was not a high priority prior to the 2014 West Africa outbreak. However, since 2014, the reality of the public health threat imposed by EBOV has been brought to the forefront. Of the 97 National Institute of Health (NIH) clinical trials for EVD, 88 were registered after 1 January 2014. Additionally, several vaccine platforms against MVD have shown pre-clinical efficacy in NHPs, including DNA vaccines, virus-like particles, recombinant adenovirus vectors, and recombinant vesicular-stomatitis virus (VSV), with multiple successful phase I clinical trials [57,58,59,60].

With the increased efforts and resources in developing a vaccine for EBOV as of 2014, there is now an FDA approved vaccine for EBOV. The vaccine produced by Merck, VSV-ZEBOV, is a live, attenuated recombinant VSV expressing the GP of *Zaire ebolavirus*. This vaccine was initially developed by the Public Health Agency of Canada (PHAC) in 2001, known as V920 during its investigational phase, and was licensed by a subsidiary of NewLink Genetics Corp in 2010. In late 2014, with the 2014 West Africa Ebola outbreak at its peak, Merck acquired the rights to develop this vaccine. V920, later trade named Ervebo, was deployed in a “ring vaccination” trial and was determined to be 100% efficacious against EVD [61]. In November 2019, the European Commission granted conditional marketing approval, and within 48 h, the WHO approved the vaccine’s prequalification. The FDA approved the vaccine in December 2019, and as of June 2020, over 300,000 people in the DRC and surrounding countries have been vaccinated. Although Ervebo received fast-track approval during the 2018 Ebola outbreak in the DRC, limiting the spread of the outbreak, the history of EVD outbreaks previously demonstrated the need to develop a vaccine against EBOV long before either the 2014 or the 2018 Ebola outbreaks. Furthermore, while EBOV has been responsible for the greatest number of infections and deaths to date, other filoviruses have demonstrated through past outbreaks that they have similar epidemic potential.

To overcome the lack of funding and resources for developing vaccines against other members of the filovirus family, alternative solutions are being investigated to combat these viruses. Monoclonal antibodies (mAbs) are an accepted treatment for infectious diseases, especially for those which lack an effective vaccine. Passive antibody administration is an effective method for providing rapid immunity to susceptible individuals against a specific pathogen as a pre- and a post-exposure prophylaxis. Although passively administering potent mAbs against infectious agents can confer protection, it is not long-lasting and requires repeat dosing for long-term efficacy. An alternative approach to the traditional passive transfer antibodies is to utilize human cells directly in vivo to “manufacture” neutralizing mAbs, by vectorizing antibody expression. With the use of highly potent, and well characterized mAbs specific for not only EBOV, but other members of the filovirus family, potential to develop a pan-filovirus cocktail of AAV-mAbs to protect individuals against future outbreaks remains untapped.

## 4. Filovirus Monoclonal Antibodies and the Potential of Antibody Therapy

### 4.1. Murine Derived Monoclonal Antibodies

The first attempt to use antibodies for passive immunization involved administration of convalescent serum, dating back as early as the 1940 [62]. Low concentrations of antibodies as well as safety concerns around convalescent serum led to the development of mAbs, providing well characterized, safer, and documented sources of pathogen-specific antibodies. An ever-increasing panel of mAbs have been investigated for Ebola virus neutralization activity in both pre- and post-exposure scenarios in a variety of animal models, including mice, guinea pigs, ferrets, and NHPs [63,64,65,66,67,68]. mAbs-based therapies have been shown to be efficient at reversing the progression of a lethal Ebola virus infection in NHPs, which closely recapitulates the disease in humans [69,70,71,72]. Past studies have demonstrated that the humoral immune response correlates with survival and plays a critical role in protection [73,74].

Earlier studies in BALB/c mice immunized with VSV pseudotyped with the EBOV GP (VSVΔG/ZEBOVGP) resulted in the generation of mAbs 1H3, 2G4, 4G7, 5D2, 5E6, 7C9, 7G4, and 10C8 [75]. These murine mAbs exhibited high-affinity binding to diverse domains on the GP protein, with some possessing neutralizing activity. mAbs 5D2, 5E6 and 7C9, which bind to the heavily glycosylated mucin-like domain (MLD) [76], and IH3, which binds the glycan cap, are non-neutralizing and likely rely on effector functions to be confered protection, while neutralizing mAbs 2G4 and 4G7 bind within the GP1-GP2 interface potentially preventing structural changes required for membrane fusion [77]. Following challenge with 1000xLD_50_ mouse-adapted *Zaire ebolavirus* (MA-ZEBOV), mAbs 5D2, 5E6, 7C9, and 7G4 provided 100% protection in mice, and neutralizing mAbs 2G4 and 4G7 conferred 60% protection in the guinea pig model [66]. ZMab, a cocktail of 1H3, 2G4, and 4G7, was able to completely protect NHPs when the first of three doses of the cocktail was administered 24 h pre-exposure to EBOV [78].

MB-003, a cocktail comprised of 13C6, 13F6, and 6D8, was generated from mice immunized with Venezuelan equine encephalitis virus replicons encoding the GP from EBOV [79]. Murine antibody variable regions were subsequently chimerized with human IgG constant regions and produced in tobacco (*Nicotiana benthamiana*) [80,81]. MB-003 mAbs were individually able to confer protection both one day prior and one day-post exposure to 300xLD_50_-MA-EBOV [81]. Furthermore, 13F6 and 6D8 bind the MLD with high affinity, despite being unable to neutralize, while 13C6 binds the glycan cap of GP but does not neutralize [79]. In an NHP study, 67% protection resulted from administration of MB-003, either 24 or 48 h post-infection with a lethal dose of EBOV with no apparent adverse effects or viremia from infection in those NHPs that survived [71].

Optimization of various mAb combinations led to the development of ZMapp, a combination of 2G4 and 4G7 from ZMab and 13C6 from MB-003 [82]. ZMapp administration five days post-exposure to EBOV challenge resulted in rescue of 100% of NHPs. High fever, viremia, and coagulation abnormalities, elevated liver enzymes, mucosal hemorrhages, and generalized petechia were developed by the NHPs prior to ZMapp administration, which was reversed, leading to full recovery [82]. During the 2014 West Africa Ebola outbreak, ZMapp was granted fast track approval from the FDA and a randomized, controlled trial of ZMapp was conducted nearing the end of the epidemic. Of the 72 individuals enrolled in the trial, there was a 30% overall case fatality rate, with 37% fatality from those who received standard care alone and 22% fatality from those who received standard care plus ZMapp [83]. Although ZMapp appeared to be more beneficial than standard care alone, the trial did not enroll a sufficient number of participants to reach statistical significance [83].

While effective, the first generation of EBOV mAbs were generated in mice, which has some disadvantages for human administration. For example, murine mAbs are swiftly degraded in human circulation as they cannot undergo antibody recycling and can cause an anti-drug antibody (ADA) humoral response [84,85]. Engineering murine mAbs to more closely resemble human antibodies, referred to as chimeric antibodies (chAbs) or humanized antibodies (huAbs), can potentially alleviate ADA immune responses [86]. huAbs are less likely to incite an immune response in humans and NHPs than murine or chimeric antibodies, making them attractive immunotherapies when therapeutics can be administered at high dosages and potentially more than once [87,88,89]; however, antibody isolation techniques have enabled identification of mAbs directly from human samples, providing even less risk of ADA.

### 4.2. Human Derived Monoclonal Antibodies

In more recent years, screening of human B cells from survivors of filovirus infection has led to the isolation and characterization of potent mAbs showing therapeutic effects in animal models as monotherapies or in cocktails [82,90,91]. Two potent mAbs, 100 and 114, were characterized from the serum of human survivors of the 1995 Kikwit outbreak more than a decade later [92]. Furthermore, 100 binds to the base of the GP, and is likely to compete with 2G4 and 4G7 of ZMapp, while mAb 114 binds in the same region of the glycan cap as 13C6 of ZMapp; however, mAb 114 is neutralizing [90]. Strikingly, monotherapy of 114 or in a cocktail with mAb 100 was 100% protective in NHPs challenged with a lethal dose of EBOV 3–5 days post infection [90]. mAb 114 was tested in a phase I clinical trial for safety, tolerability, pharmacokinetics, and immunogenicity, showing no adverse reactions at the time of injection, 22% of individuals reporting mild reactogenicity within the first four days, and no long-term solicited adverse events post injection in the participants [93]. In a randomized, controlled phase II clinical trial, mAb 114 was compared against the mAb cocktails ZMapp and REGN-EB3 (invented by Regeneron) as well as remdesivir, a broad-spectrum antiviral. After the enrollment and administration to 681 consenting laboratory-confirmed EVD patients, it was recommended based on the positive results of the interim analysis that patients be assigned only to the MAb114 and REGN-EB3 groups for the remainder of the trial [83]. Though proven protective against EVD, both mAb Mab114 and REGN-EB3 protection does not extend to other members of the filovirus family.

As with EBOV, MARV GP-specific neutralizing antibodies have been shown to reduce mortality after challenge with a lethal dose of MARV [69]. One of the many antibodies isolated from a human survivor of MARV (Table 1), MR191 protected NHPs challenged with a lethal dose of MARV when administered intravenously either 4 or 7 days post challenge [94]. MR191 neutralizes MARV through competing with Niemann–Pick C1 (NPC1) for the receptor-binding site (RBS) on MARV [95]. Additionally, some of these MARV mAbs have the ability to cross-react with EBOV GP. MR78, MR111, and MR191 bind EBOV GP lacking the glycan cap and MLD, while MR72 binds the full EBOV GP as well as variants [95]. Although seven clinical trials investigating various methods of treatment and prevention of MVD have been registered with clinicaltrials.gov, none of these studies have evaluated the use of mAbs [58,59,60,96].

### 4.3. Pan-Ebolavirus Monoclonal Antibodies

Since the 2014 West Africa Ebola outbreak, an increase in highly potent mAbs have been isolated and characterized, with some providing pan-ebolavirus protection (Table 2) [99]. This class of antibodies shows cross-species binding of GP and in some cases neutralizes multiple filoviruses. CA45, an mAb developed through immunizing NHPs with a trivalent GP cocktail (EBOV/SUDV/MARV GP_Δmucin_), generated antibodies that bound and neutralized pseudotyped VSV displaying EBOV, SUDV, or BDBV GP [99]. CA45 binds the internal fusion loop of the N-terminus of GP and rapidly neutralizes EBOV, SUDV, BDBV, and RESTV [100]. CA45 targets a conserved epitope at the base of the GP in close proximity to EBOV-specific mAbs, 2G4 and 4G7 [100]. However, CA45 binds at a slightly different angle, allowing for better binding to both GP_1_ and GP_2_, partially inhibiting GP cleavage at an earlier step in viral fusion as well as blocking the virus post GP cleavage. Furthermore, CA45 alone or in combination with FVM04 provided full protection at a low dose (10 mg/kg) against EBOV, SUDV, and BDBV in mouse, guinea pig, and ferret animal models [100,101]. FVM04 in combination with 13C6 and 2G4 demonstrated increased survival in a lethal guinea pig adapted (GPA)-EBOV challenge compared to ZMapp, and extended survival against GPA-SUDV challenge, demonstrating the cross-protective nature of this antibody cocktail [101].

ADI-15878 is a potent neutralizing mAb isolated from a survivor of the 2014 West Africa Ebola outbreak that binds the highly conserved region of GP_2_, locking GP into a pre-fusion state as well as targeting a cleaved intermediate prior to viral fusion [102]. ADI-15878 provided 100% protection in mouse models of EBOV and SUDV infection; however, it was only partially protective against EBOV and SUDV in guinea pig and ferret models [102,103,104]. ADI-15946 is another potent pan-ebolavirus antibody that neutralizes VSV pseudotyped with GPs from EBOV, BDBV, and SUDV. ADI-15946 was specificity-matured for SUDV GP binding affinity using yeast-display technology and the resultant variant, named ADI-23774, was combined with ADI-15878 to form the pan-ebolavirus cocktail MBP134. MBP134 potently neutralized EBOV, BDBV, SUDV, TAFV, RESTV, and BOMV pseudotyped VSVs and protected 70–100% of guinea pigs five days post-challenge with GPA-SUDV [104]. MBP134 demonstrates limitless engineering and advancements of filovirus-specific mAbs, allowing for the potential development of a potent pan-filovirus mAb cocktail.

## 5. AAV-Mediated Monoclonal Antibody Expression for Filovirus Infections

### 5.1. AAV-Mediated Monoclonal Antibody Expression

**Figure 1:**
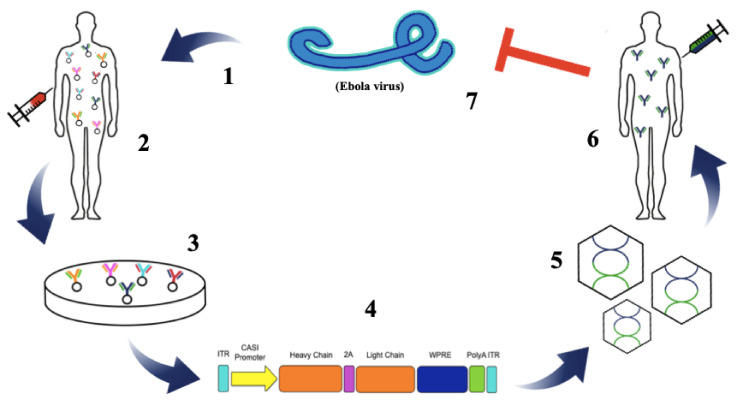
Schematic representation of adeno-associated virus vectored-immunoprophylaxis (AAV VIP) against Ebolavirus. Ebolavirus infects a healthy patient causing the onset of Ebola hemorrhagic fever (EHF) (**1**). B cells are isolated from the blood of an Ebola virus disease (EVD) survivor (**2**). Potent antibodies are isolated for further development and characterization (**3**). Variable-heavy and variable-light chains of selected mAbs are cloned into the AAV vector (**4**). AAV vectors expressing the potent mAb against ebolavirus are manufactured (**5**). Individuals susceptible to ebolavirus infection are intramuscularly administered AAV-mAb vector (**6**). mAbs are secreted from the AAV-mAb transduced muscle cells into the blood stream and circulate throughout the blood, protecting against ebolavirus infection (**7**).

FDA approval of AAV-based gene therapy products Luxterna (voretigene neparovec) and Zolgensma (onasemnogene abeparvovec) has dramatically accelerated the clinical progression of AAV therapeutics [109,110]. AAV vectors have an established record of high-efficiency gene transfer in a variety of model systems [111]. When delivered to post-mitotic organs including muscle, brain, and liver, AAV vector genomes assume the form of intranuclear high-molecular weight episomal concatamers that direct transgene expression for extended periods of time [112,113]. Vectored immunoprophylaxis (VIP), a term first coined by David Baltimore and colleagues [114], is used to describe AAV-mediated delivery of mAb genes (Figure 1). AAV mAbs are typically expressed as full-length antibodies with heavy and light chain sequences separated by an F2A self-cleaving peptide containing a furin recognition sequence between the heavy chain gene and 2A, which allows for removal of 2A residues from the upstream gene [114,115]. Administration of AAV expressing genes encoding well characterized pathogen specific mAbs leads to continuous and sustained secretion of antibodies into the blood stream resulting in protection against a wide range of potential infectious agents [116]. This strategy has been shown to be highly effective at protecting mice against both systemic and mucosal HIV infection as well as protecting rhesus macaques against systemic simian immunodeficiency virus (SIV) infection [114,117,118]. In these studies, AAV expressed mAbs were detected in the circulation at therapeutic levels for the lifetime of the host after only a single IM injection [114]. Delivery of AAV-encoded HIV mAbs 10-1074, 3BNC117, and 10E8 to rhesus macaques that had been infected with SHIV-AD8 for 86 weeks prior to receiving AAV therapy showed proof of concept that AAV-delivered mAbs have the potential to provide a functional cure for HIV [119]. Evaluations of bNAb levels after AAV administration revealed that 10E8 was low or undetectable in all animals, 3BNC117 was delivered successfully in one out of four animals and 10-1074 achieved significant expression levels in three out of four NHPs. Although ADA responses led to a decline in mAb expression, one monkey successfully maintained 50–150 μg/mL of 10-1074 and 3BNC117 for more than two years and controlled SHIV viremia for over three years, demonstrating the power of VIP [119]. In a recent report, a macaque that received a single injection of an AAV vector encoding the anti-SIV antibody 5L7 achieved high levels of serum 5L7 IgG1 expression (~240–350 μg/mL) for over six years and this conferred sterile protection against six successive, escalating dose, intravenous challenges with highly pathogenic SIVmac239, including a final challenge with 10 animal infectious doses [120]. Importantly, this NHP generated little or no ADA to the AAV-delivered mAb for the duration of the study.

While the initial application of VIP was for the prevention of HIV, subsequent infectious disease applications have been explored including influenza virus, malaria, and HCV [114,117,121,122,123,124,125] among others. For example, AAV vectors encoding broadly neutralizing influenza antibodies demonstrated protection in mice against multiple influenza virus strains following a single IM injection and this protection was sustained one year after AAV administration [121]. Notably, immunodeficient and aged mice as well as ferrets were protected by this method, suggesting that AAV-mediated mAb expression is sufficient to protect from influenza-induced illness in a variety of animal models [126]. This broad range of successful pre-clinical applications justified the expansion of this platform to target filovirus diseases where potent neutralizing mAbs have previously been isolated and characterized as reviewed above.

### 5.2. Pre-Clinical Models of AAV-Mediated Monoclonal Antibody Expression and Protection against Filovirus Infections

Currently, a limited number of groups have published data on AAV-mediated expression of mAbs for the treatment and prevention of filovirus infections. Although this is not a traditional vaccine approach, the goal of employing AAV-mediated mAb expression for filoviruses is to generate long-term immunity for populations at risk of being exposed to the virus during outbreaks. The first group to evaluate this approach utilized AAV9 to express murine mAbs, 2G4, 4G7, and 13C6, which were previously successful in the treatment of EBOV in macaques [78,127]. Following intranasal administration of vector, mice were protected from a lethal challenge of EBOV, while intramuscular administration was hampered by systemic immune responses against the chimeric mouse/human antibodies. Humanizing the mAbs in the cocktail resulted in sustained mAb expression and a two-fold increase in mAbs in the serum and bronchoalveolar lavage fluid [128]. This group indicated that intravenous administration may potentially result in higher transgene expression than traditional intramuscular routes of VIP administration. Additionally, they indicated that an antibody-specific humoral response limits protective efficacy, indicating that capsid and antibody gene selection is critical to minimize humoral immune responses for successful VIP therapeutics. Our group utilized a novel AAV6 variant, termed AAV6.2FF, to deliver 2G4, 5D2, and 7C9 mAb genes to the muscle demonstrating that non-neutralizing mAb VIP monotherapies alone can confer 100% protection against lethal EBOV infection in mice [66,116]. A minimum of seven days lead time was required for VIP administration to provide 100% protection from lethal EBOV challenge in mice, indicating that this approach may provide protective immunity more rapidly than traditional vaccinations. Finally, AAV-mediated mAb expression from a single administration given five months prior to lethal challenge with MA-EBOV protected 100% of the mice demonstrating the potential of this VIP platform to be used as an alternative vaccination strategy for filovirus diseases. Ultimately, the VIP approach to filovirus prevention still requires optimization, but currently represents a promising area for filovirus disease prevention.

### 5.3. Clinical Relevance

Many late-stage clinical trials are currently underway studying the effects of AAV-based gene therapy on a variety of genetic diseases including alpha-1 antitrypsin deficiency and hemophilia B [129,130,131]. The safety profile and efficacy shown in the outcomes of these trials among others have paved the way for many AAV therapies to enter the clinic including those that are using VIP for the prevention and treatment of HIV [124]. Promising results in animal models of SIV and HIV following rAAV administration encoding broadly neutralizing antibodies and immunoadhesins have driven AAV-mediated mAb producing therapies forward into clinical evaluation as an alternative to long-term recombinant mAb re-administration [132]. One of the current clinical trials involves AAV-mediated antibody gene delivery for the prevention of HIV infection (NCT01937455) using rAAV1 to deliver mAb PG9 [124]. PG9 was previously found to be highly potent, able to neutralize a broad panel of HIV strains, and achieved 90% inhibition of a diverse set of HIV-1 viruses at concentrations far lower than competing broadly neutralizing antibodies. During the course of the trial, a singular mild adverse event due to the treatment regimen suggests that the use of AAV-mediated gene transfer represents a safe method of therapeutic delivery in healthy individuals that does not differ from the safety profile of a preventative vaccine [124]. However, very low PG9 expression was observed, suggesting that the AAV serotype, expression strategy, antibody selection, and immune response mitigation must be optimized for future studies. Another phase I clinical trial is underway (NCT03374202) examining the delivery of a potent broadly neutralizing HIV antibody, VRC07, utilizing AAV8 for targeted gene delivery to the muscle in HIV patients using a vector system that differs minimally from those outlined in the filovirus VIP pre-clinical study data [117]. The outcomes of these clinical studies for HIV will inform future platform development for filovirus VIP therapies [110,133].

### 5.4. Benefits of the AAV VIP Approach

In vivo delivery of mAb genes for pre-or post-exposure prophylaxis offers an alternative to conventional vaccines and passive immunization. With the VIP, there is little need to understand complex correlates of immunity as in vaccine development; one simply needs the sequence of a protective mAb which are often available well before effective vaccines. AAV-mAb expression provides less expensive and longer lasting immunity than passive immunization and would be a useful tool in the event of a filovirus virus outbreak, particularly for ring vaccination purposes. VIP circumvents the lead time required for vaccine induced adaptive immunity to mount before being effective. Since AAV-mAb expression results in production of protective antibodies independent of the immune system, it can be employed to protect individuals who do not respond to traditional vaccination or who are lacking a properly functioning immune system, such immunocompromised, elderly or pediatric populations. VIP is an ideal stop gap measure for intervention in outbreaks where a vaccine is unavailable (e.g., MARV and SUDV) and could provide a synergistic effect in combination with a traditional vaccine to combine the benefits of both immunization strategies. Moreover, AAV is a remarkably stable vector that can be lyophilized to facilitate long-term stockpiles as well as formulated to negate cold chain requirements for transportation [134].

### 5.5. AAV VIP Challenges and Potential Solutions

Although AAV vector-mediated delivery of mAbs holds great promise for treating and preventing infectious diseases that lack effective vaccines, including filovirus infections, there are a number of potential hurdles that still need to be addressed. For example, in NHP models of AAV VIP for HIV [118,120,135,136], as well as in the first human clinical trial in which AAV was used to express the broadly neutralizing HIV mAb, PG9 [124], ADAs were detected and this correlated with loss of mAb expression. However, broadly neutralizing HIV Abs (bNAbs) appear after several years of infection and are known to exhibit an unusually high rate of somatic hypermutation with frequencies of up to 32% in the heavy chain variable region and extended complementarity-determining regions [137,138,139]. In contrast, the number of mutations observed in influenza antibodies rarely exceeds 10% and in the case of VSV, early primary response VSV-neutralizing IgGs are free of somatic mutations [140,141]. Currently, it is unknown whether such highly mutated regions in HIV bNAbs make them targets of anti-drug antibody responses or whether the same would occur with mAbs with fewer somatic mutations. It would be interesting to compare AAV-mediated gene transfer efficacy and the extent of ADA formation against an HIV bNAb such as PG9 or 10E8 and a filovirus antibody such as MR191 in a large animal model since up to this point, the only AAV-mAbs that have been evaluated in NHPs have been broadly neutralizing HIV or SIV mAbs [95,142,143,144]. Finally, a recent SHIV-AD8 challenge study in macaques found that the formation of ADAs was more pronounced when AAV-expressed mAbs were of the IgG1 isotype, which has been the default isotype for AAV VIP, rather than IgG2, suggesting that optimization of the mAb isotype may limit the formation of ADA [145].

Host immune responses to the AAV vector itself have been shown to limit the ability to attain therapeutic concentrations of AAV expressed mAbs. In the first human clinical trial involving AAV VIP, both anti-AAV1 capsid antibodies and AAV1-capsid specific T-cell responses were detected [124]. Recently, systemic co-administration of AAV vectors with poly(lactic acid) (PLA) nanoparticles containing rapamycin (SVP(Rapa)) was shown to efficiently block the induction of both antibody and T cell anti-capsid responses in an antigen (i.e., AAV capsid) specific manner [146,147,148]. Pre-existing neutralizing antibodies to the AAV capsid may also reduce the efficacy of AAV VIP therapies; however, a recent report by Elmore and colleagues describes a clinically relevant strategy to rapidly and transiently degrade neutralizing AAV capsid antibodies prior to AAV administration [149,150,151,152]. In this case, a single intravenous dose of the IgG-degrading enzyme, IdeZ, rescued AAV transduction by transiently reversing seropositivity, mitigating the effects of preexisting anti-AAV NAbs in mice and NHPs [153]. Finally, engineering of novel AAV variants with reduced neutralizing antibody recognition is another approach being used to circumvent preexisting humoral immunity to AAV vectors [154,155].

In some circumstances, such as in pediatric patients with rapid tissue growth, re-administration may be required. While some serotypes of AAV can be efficiently re-administered if the time between administrations is longer than 28 weeks [156,157], a number of studies have reported successful re-administration after pretreatment of the host with immunosuppressing antibodies [158,159,160,161], for example, B cell depleting monoclonal antibody, rituximab [162,163], immune-modulating agents [164], or broad immunosuppressing agents, such as cyclophosphamide [165].

The ability of AAV vectors to achieve long-term transgene expression has been attributed to their relatively low immunogenicity [166,167,168]; however, AAV vectors have been shown to activate various Toll like receptors (TLRs) and this can have an influence on the outcome of gene transfer [169,170]. In particular, the AAV DNA genome and capsid are sensed by TLR9 and TLR2, respectively [171,172], and AAV vectors carrying double-stranded genomes show higher immunogenicity relative to those carrying single-stranded genomes [173]. Recent findings also suggest that CpG sequences in the AAV genome contribute to transgene immunogenicity but that CpG-depletion of AAV vectors can promote persistent transgene expression and minimize infiltration of effector cells that target AAV-transduced cells [173,174]. Although these observations were made using preclinical animal models, factors such as CG dinucleotides that influence transgene immunogenicity should be carefully evaluated as part of the clinical translation pipeline.

Lastly, apart from the waning of mAb expression due to natural turnover of AAV transduced cells, there are currently no FDA-approved methods for turning off expression of AAV-mAbs once administered, should there be a need. Developing a “kill-switch”, where administration of a protein could inhibit further expression of AAV-mAbs would be an added safety measure and may allow for the application of short-term AAV VIP therapies [175]. Finally, though AAV VIP may offer more sustained and consistent mAb expression than traditional passive immunization, the cost of producing AAV vectors is not insignificant.

The next few years should provide improvements to AAV mAb gene delivery that will ultimately reveal whether AAV VIP is a viable platform for expressing therapeutic levels of protective mAbs in both preventive and therapeutic contexts.

## 6. Future Directions

The concept of VIP was first established in 2002 [125]; however, it is only more recently that this approach has been expanded to infections beyond HIV and clinical evaluation has begun [125]. Further vector design improvements to maximize mAb expression including selection of regulatory elements in the vector genome (i.e., promoter, poly adenylation signal) and antibody expression sequence (i.e., leader peptide, codon optimization) as well as targeting specific cell types of interest through capsid engineering or tissue specific promoters will enable indication-specific optimization of the VIP platform.

We and others have demonstrated AAV-mediated expression of mAbs against Ebola virus to be an effective method of protecting mouse models from lethal infection. To date, these studies have focused on EBOV specific antibodies; however, there are a number of more recent publications highlighting the availability of mAbs able to bind and neutralize multiple species of *Ebolavirus* including EBOV, SUDV, and BDBV, which will enable the development of pan-ebolavirus mAb cocktails [100,103,104,108]. Furthermore, mAbs that bind to both EBOV and MARV have been identified creating the possibility of pan-filovirus mAb therapies, although cross-neutralizing antibodies for both these filoviruses remain elusive [71,95,176].

The antibody engineering field has generated dozens of innovative designs for therapeutic antibodies to facilitate a variety of desired functions [177]. The packaging capacity of AAV facilitates the generation of vectors expressing bispecific antibodies such as dual variable domain immunoglobulins (DVD-Igs) [178]. Conversely, multidomain antibodies string together a number of single domain antibodies or nanobodies to facilitate binding to numerous epitopes while DVD-Igs are limited to two [179]. Both of these strategies enable the expression of multiple antigen binding fragments, which establishes multivalent targeting of diverse species or epitopes using a single vector. In the context of filoviruses, bispecific or multidomain antibodies in combination with pan-filovirus activity could result in a VIP product capable of providing protection against all filovirus infections in a single product.

Here we reviewed the VIP platform with a focus on filoviruses; however, there are a number of parallels with other infectious diseases affecting populations in poverty with poor health care infrastructure, insignificant funding for research and development of prophylactics or therapeutics and a lack of licensed vaccines, which is still the case for all but one of the pathogenic filoviruses despite more attention in recent years. The aim of the VIP platform is to confer long term expression of protective mAbs to facilitate immunity against infection, whether the target is a filovirus, HIV, or any other infectious disease of public health importance, the methodology is the same and learnings from one indication can be applied to new pathogens in the future.

## Figures and Tables

**Table 1:** Top performing mAbs for MARV from human survivors.

	Antibody and Source	Target	Competes with	Cross-Binds	Cross Neutralizes	Protection	Reference
MR72	Human IgG1; Survivor from 2014 MARV outbreak in Uganda	Receptor-binding site (RBS) near the hydrophobic trough and the apex of GP1	MR78, MR82, MR191	MARV (all strains), EBOV GP	MARV (Uganda), RAVV	100% protective in mice (n = 5) against MARV (Uganda) in mice	[95]
M78	Human IgG1; Survivor from 2014 MARV outbreak in Uganda	Binds to the top and side of GP1 at a shallow angle relative to the central three-fold axis	MR72, MR82, MR191	MARV (Uganda) and RAVV	MARV (Uganda and Ravn), potentially neutralizes EBOV entry by inhibiting viral membrane fusion downstream from virus receptor recognition	100% protective in guinea pigs against MARV (Angola) and 100% against RAVV	[95,97]
MR82	Human IgG1; Survivor from 2014 MARV outbreak in Uganda	Binds toward the top and side of GP1 at a shallow angle relative to the central three-fold axis	MR72, MR78, MR191	MARV (all strains)	MARV (all strains)	40% protective in guinea pigs; however, it was 100% effective in mice	[95]
MR191	Human IgG1; Survivor from 2014 MARV outbreak in Uganda	RBS near the hydrophobic trough and the apex of GP1	MR72, MR78, MR82	MARV (Muskoke and Angola), RAVV, EBOV GP (does not neutralize)	MARV (Angola and Muskoke)	100% protective against MARV (Angola) and RAVV in guinea pigs. 100% effective in NHPs when treated on day 4 and 7 post infection (MARV Angola). 80% protective on days 5 and 8 post infection of RAVV. Did not protect against GPA-SUDV in guinea pig models 3 dpi	[94,95,98]

**Table 2:** Top performing pan-Ebola mAbs from human survivors.

	Antibody and Source	Target	Competes with	Cross-Binds	Cross Neutralizes	Protection	Reference
CA45	Macaque; Immunized macaque against EBOV, SUDV and MARV GPs	Conserved epitope in the internal fusion loop. Binds to the native GP but binds with way higher potency to the cleaved GP. Does not bind soluble GP (sGP)	KZ52, 2G4 and 4G7	EBOV, SUDV and BDBV (does not bind MARV, Tai Forest or LLOV)	Potently neutralizes EBOV, SUDV, and BDBV and moderately neutralizes Reston. IC50 for EBOV and cleaved core ectodomain of EBOV (EBOV GPcl) was 4.63 and 0.05 nM, BDBV and GPcl was 4.24 and 0.007 nM, and SUDV and GPcl was 9.16 and 0.05 nM	In mice (n = 20), protection was as good as against EBOV and 80% against SUDV. In guinea pigs (n = 6) it was 100% protective against EBOV and SUDV. 100% protection in ferret model (n = 4) of BDBV. FVM04 + CA45 in mice against SUDV was 100% effective, mirrored in guinea pigs for EBOV	[100]
BDBV223	Human IgG3; Human survivor of 2007 Uganda BDBV outbreak	GP2 stalk (canonical heptad repeat 2 (HR2) domain near the MPER) (71% conserved amongst the five first Ebolaviruses, and 90% conserved amongst EBOV, BDBV, and SUDV), does not recognize BDBV sGP	BDBV317, BDBV340	rVSV-GP of EBOV, BDBV, and SUDV	rVSV-GP of EBOV, BDBV, and SUDV	100% in mice (n = 5), post-exposure (1 day) was 20% protection against lethal EBOV (Mayinga) challenge in guinea pigs (n = 5) and 50% effective in ferret models (n = 4), 3- and 6-days post-challenge	[104,105,106]
FVM04	Cloned as human IgG1 with kappa light chain; Macaque vaccinated with EBOV, SUDV, and MARV GP	The exposed tip of the receptor binding domain crest at the apex of the GP trimer and blocks interaction of GP with NPC1 in the late endosome. Glycans may moderately interfere with optimal binding. The RBD crest is highly conserved	M13C6, partially KZ52	EBOV, SUDV, and BDBV (Binds WT, cleaved and sGP of all Ebola species), low affinity binding to MARV and Reston	EBOV and SUDV but not really BDBV or MARV. It is a weaker neutralization than some others: EC50 of 4.3 and 4.3 ug/mL for SUDV and EBOV	100% in mice against EBOV and 75% in mice against SUDV. 100% protective in guinea pigs against SUDV and 40% protective against EBOV	[101,107]
ADI-15742	Human IgG; Human survivor of 2014 West Africa outbreak	A highly conserved region of GP2 within the internal fusion loop. Locks the GP into a pre-fusion state whether free or bound to NPC1. Does not bind sGP	ADI-15878, 100, KZ52, CA45	Everything but MARV/LLOV (rVSV-GP for each Ebolavirus and Marburgvirus)	Potently neutralizes all five Ebola virus with IC50 of <2 nM for rVSV pseudotypes	100% protection in lethal mouse model (100 μg against 100 pfu MA-EBOV) The two ADIs are clonal siblings with 90% and 95% chain identity. *	[102]
ADI-15878	Human IgG; Human survivor of 2014 West Africa outbreak	A highly conserved region of GP2 within the internal fusion loop. Locks the GP into a pre-fusion state whether free or bound to NPC1. Does not bind sGP. Seems to target a cleaved intermediate in endosomes while first generation base binders act further upstream	100, KZ52, CA45	Everything but MARV/LLOV	Potently neutralizes all five Ebola virus with IC50 of <2 nM for rVSV pseudotypes	80% protective in mice (n = 10) against EBOV and 100% protective in mice (n = 20) against SUDV. 75% protective against BDBV in ferrets (n = 4)	[102]
ADI-23374	Derived from affinity maturation of a human mAb (Human survivor of 2014 West Africa outbreak ADI-15946) using yeast resulting in enhanced potency against SUDV.	ADI-15946 binds a highly conserved epitope shielded by the mobile β17–β18 loop of the glycan cap, which is where ADI-23774 binds, does not bind sGP of EBOV	EBOV, BDBV, SUDV	EBOV, BDBV, SUDV	rVSV-SUDV GP	90% protective against EBOV in mice (n = 10) 3 days post exposure and was 100% protective against wt-SUDV in mice (IFNR-/- mice) (n = 10) at 1- and 4-days post exposure	[108]

* They appear to bind the exact same location, although ADI-15878 engenders viral neutralization escape to a lesser extent than ADI-15742.
